# The Current Status of Heterogeneous Palladium Catalysed Heck and Suzuki Cross-Coupling Reactions

**DOI:** 10.3390/molecules23071676

**Published:** 2018-07-10

**Authors:** Philani P. Mpungose, Zanele P. Vundla, Glenn E. M. Maguire, Holger B. Friedrich

**Affiliations:** 1 Catalysis Research Group, School of Chemistry and Physics, University of KwaZulu-Natal, Westville Campus, Durban 4000, South Africa; 207506038@stu.ukzn.ac.za (P.P.M.); sjaejj@gmail.com (Z.P.V.); 2 School of Chemistry and Physics, University of KwaZulu-Natal, Varsity Drive, Durban 4001, South Africa; maguireg@ukzn.ac.za

**Keywords:** C–C cross coupling, Heck cross-coupling, Suzuki cross-coupling, heterogeneous palladium catalysis

## Abstract

In the last 30 years, C–C cross coupling reactions have become a reliable technique in organic synthesis due their versatility and efficiency. While drawbacks have been experienced on an industrial scale with the use of homogenous systems, many attempts have been made to facilitate a heterogeneous renaissance. Thus, this review gives an overview of the current status of the use of heterogeneous catalysts particularly in Suzuki and Heck reactions. Most recent developments focus on palladium immobilised or supported on various classes of supports, thus this review highlights and discuss contributions of the last decade.

## 1. Introduction

The importance of carbon–carbon bond forming reactions is well documented in literature [1,2,3]. The industrial importance of Suzuki and Heck cross-coupling reactions along with numerous others in this general field has sparked great interest in C–C bond formation reactions. In addition, the lack of a universal set of reaction conditions for every catalyst system and the drawbacks of industrially employed catalyst systems continue to fuel this interest. Heck and Suzuki reactions are typically catalysed by palladium based homogenous systems that have illustrated high turnover frequency (TOFs). These homogeneous catalysts require the use of ligands (phosphine or *N*-heterocyclic) to form active catalysts. As a result, separation of these catalysts (the ligand, the Pd metal or the complex) from the reaction media has presented the greatest challenge in this field. Consequently, the catalyst gets incorporated into the final product, resulting in loss of catalyst and a devalued product especially since these reactions are at the forefront of the production in the pharmaceutical industry. Successful separation of these homogeneous catalysts from the product solution requires the use of expensive nanofiltration membranes or an extensive (and usually destructive) column chromatography separation. Table 1 summarises the maximum limits of residual metal catalysts acceptable in the pharmaceutical industry.

These low allowed concentrations have prompted the development of recoverable and reuseable catalyst systems to decrease product contamination and alleviate the loss of catalyst. Many attempts have been made over the years to heterogenise homogeneous systems and to develop heterogeneous systems to displace homogenous systems and this review aims at highlighting recent contributions of the last decade at achieving this.

The main challenge in the development of heterogeneous catalytic systems for C–C cross-coupling reactions has been in establishing the nature of the active catalyst species. While literature on homogeneous systems is well developed with the mechanism well understood, the contrary is observed for heterogeneous catalysts. The true nature of the active catalyst when a palladium containing solid material is employed as a catalyst is still a highly debated issue in the field of cross-coupling reactions [5]. In most research papers, the “true nature” of the active form of palladium is ambiguous; claims exist in the literature supporting both soluble molecular and nanoparticle catalysts, as well as truly heterogeneous insoluble Pd catalysts [5,6,7]. More specifically, the question is whether the oxidative addition of the aryl halide (Ar–X) occurs on the surface of the solid catalyst (heterogeneous catalysis) or on leached metal atoms (homogeneous catalysis). The literature is currently divided on this matter; some researchers recognise the solid pre-catalyst as a “reservoir” of soluble catalytically active palladium species [4,8,9,10]. Others claim to have developed truly heterogeneous systems, with catalysis taking place on the surface of the solid palladium based heterogeneous catalyst [11,12].

Common heterogeneous cross-coupling catalysts mainly differ in: (i) chemical nature (organic, inorganic, or hybrid organic-inorganic) of the solid matrix entrapping the Pd catalyst and (ii) the nature of the catalyst attachment (chemical or physical entrapment) [13]. There are also examples of efficient catalysts for cross-coupling reactions based on colloidal palladium particles. The usefulness of the developed catalytic system is usually determined by its activity, selectivity and the life-time of the catalyst [14]. The catalytic activity is essentially a measure of percentage of reactants that are converted to product(s); while the catalytic selectivity is a measure of the percentage of the reactants that are converted to useful or desired product(s). The life-time of a catalyst is the time that the catalyst will maintain the required level of activity and selectivity [14]. The life-time of the catalyst can also be estimated by the number of times it can be recovered and reused. Lastly, the efficiency of a catalytic process is measured in turnover number (TON) and turnover frequency (TOF).

In the following discussion, the catalytic properties discussed above will be used to determine the efficiency of the selected examples of most common palladium catalyzed heterogeneous catalytic systems for Heck and Suzuki cross-coupling reactions. The aim of the discussion is not to be exhaustive but rather to report on recent and noteworthy research progress aimed at developing heterogeneous palladium based catalytic systems.

## 2. “Naked” Palladium Nanoparticles as Pre-Catalysts

**Scheme 1:**
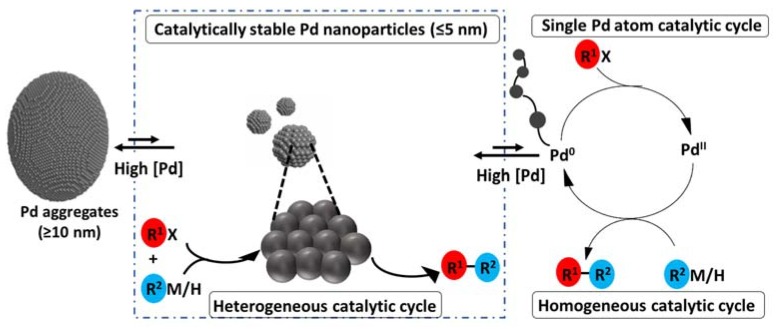
Possible reaction pathways operating in Pd nanoparticle catalysed Suzuki and Heck cross-couplings [12] (Adapted with permission from American Chemical Society).

Palladium nanoparticles have become one of the most interesting forms of heterogeneous catalysts because of their size- and shape-dependence, as well as their efficient catalytic activities in cross-coupling reactions [13,15]. The “naked” palladium nanoparticles are the simplest form of heterogeneous catalyst for C–C cross-coupling reactions. However, their use has been limited by the lack of efficient separation procedures; available separation techniques such as filtration and centrifugation are not very effective in achieving complete recovery of the nanoparticles. Additionally, nanoparticles are susceptible to oxidation, resulting in complicated work up procedures in the attempt to avoid deactivation. Furthermore, nanoparticles are also prone to agglomeration or sintering upon heating, leading to formation of insoluble non-catalytic palladium black [13]. Scheme 1 shows that a palladium metal aggregate can serve as catalytically active palladium reservoir. In situ, the palladium metal aggregates produce kinetically stable palladium nanoparticles that facilitate the heterogeneous catalytic cycle. However, single palladium atoms usually leach out from the surface of the palladium nanoparticle and participate in the homogeneous catalytic cycle.

Alternatively, supported palladium nanoparticles as catalyst for C–C cross-coupling reactions are being developed. Most often, palladium nanoparticle become less catalytically active upon immobilisation on a solid support. Therefore, an improved catalytic system with desirable aspects of both systems (high activity and easy recovery) is needed. It is envisaged that better understanding of the reactivity of palladium nanoparticle can lead to the design of more efficient heterogeneous catalyst for C–C cross-coupling reactions.

## 3. Palladium Nanoparticles on Carbonaceous Supports

**Scheme 2:**
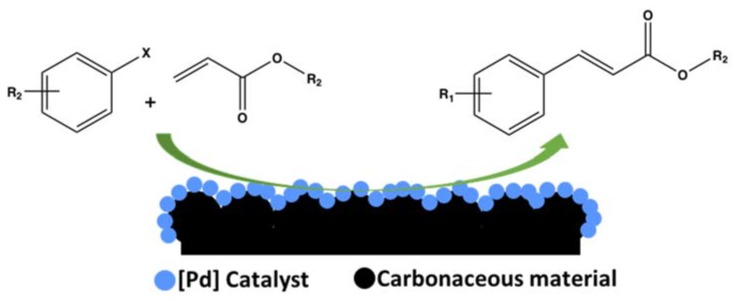
Schematic illustration of palladium nanoparticles supported on carbonaceous material as efficient catalyst for C–C cross-coupling reactions [32] (Adapted with permission from John Wiley and Sons).

Palladium supported on carbonaceous supports has been widely applied in C–C cross-coupling reactions (Scheme 2) [16,17,18]. Amongst these, palladium on activated carbon (Pd/C) is by far the most frequently used catalyst in heterogeneous Pd-catalyzed coupling reactions. This is because of its efficiency and commercial availability [16,17,18]. It can be purchased in various quantities with a palladium content ranging from 1–20%. In addition, charcoal as a solid support ensures a higher surface area compared to the corresponding alumina and silica supported catalysts [18]. Palladium on activated carbon is reported to be stable in air and water, acids and bases, and it often doesn’t require reactions to be performed under an inert atmosphere [18,19]. As a result, Pd/C catalyzed Heck and Suzuki cross-coupling reactions have been performed under a variety of reactions conditions, including in organic media [20,21,22,23], aqueous media [24,25,26], and under microwave conditions [27,28,29,30,31]. Generally, the Pd/C catalyst can convert a large variety of substrates with good to excellent yields, and it is recoverable and recyclable over many cycles [16,17,18].

**Figure 1:**
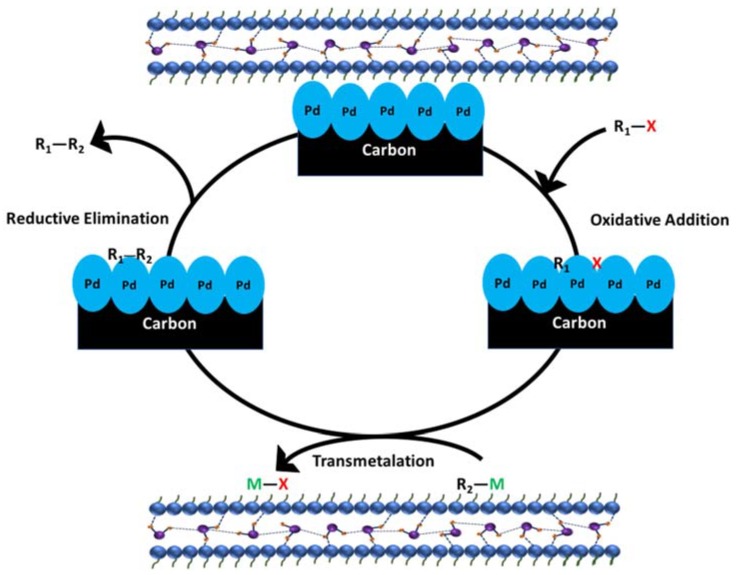
Plausible mechanism of the C–C bond forming reactions in the presence of a hydrotrope (sodium xylene sulphonate solution) [33] (Adapted with permission from The Royal Society of Chemistry).

Jadhav et al. recently (2016) developed a greener, cost effective and operationally convenient Pd/C based catalytic system for Suzuki and Heck cross-coupling reactions [33]. In their setup, the Pd/C catalyst was dispersed in an aqueous hydrotropic medium (sodium xylene sulphonate solution) that had surfactant-like properties and thus stabilised the Pd/C catalyst (Figure 1). The hydrotropism allows for easy solubility of several types of organic functionalities in water, without the need for toxic and volatile organic co-solvent. In addition, good to excellent yields of the desired product were obtained and the catalytic system was recycled three times without any significant loss in activity. Lastly, they reported that the palladium metal was “not leached” out into the product solution. Hence, this system is highly desirable from economical as well as environmental points of view.

**Scheme 3:**
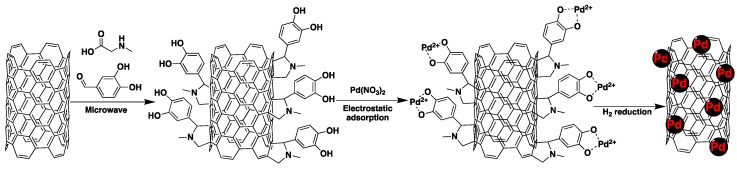
Graphical representation of Pd nanoparticles supported on carbon nanotubes [34] (Adapted with permission from Springer Nature).

Carbon nanotubes (CNTs) have emerged as highly efficient supports for palladium and they have been extensively used as alternatives to activated carbon supports [12,32,34,35]. The CNTs can be evenly distributed in solution due to their small size, thus increasing interaction between the reactants and the catalyst. In most cases, the activity of CNTs in C–C cross-coupling reactions depends on their preparation method [35]. In a recent review, by Labulo et al., a wide range of surface functionalization techniques for carbon nanotubes that improve their properties as catalyst supports was reported [34]. The resulting CNTs catalysts displayed superior catalytic performance and better recyclability in C–C cross-coupling reactions. Scheme 3 shows the graphical representation of surface functionalization and palladium nanoparticle loading onto carbon nanotubes.

Lakshminarayana et al. [32] extended the study by investigating the influence of various carbonaceous materials as supports in C–C cross-coupling reactions. They prepared and studied palladium oxide (PdO) nanoparticles impregnated on nanocarbon supports, such as single walled carbon nanotubes (SWCNT), multiwalled carbon nanotubes (MWCNT), carbon nanofiber (CNF), graphene oxide (GO), and reduced graphene oxide (RGO) [32]. The prepared catalysts were characterized fully and then explored in Heck cross-coupling reactions to assess their catalytic activity. Graphene oxide (GO) was found to be the best support for PdO (Table 2). The enhanced catalytic activity of PdO/GO was believed to be due to the combined effect of a high degree of surface-bound oxygenated moieties, high surface area, electron conductivity and the mesoporous nature of graphene oxide.

## 4. Palladium Nanoparticles on Metal Oxide Supports

A significant number of researchers have devoted themselves to finding a new and a more efficient Pd/M_x_O_y_ based heterogeneous catalyst for C–C cross-coupling reactions [36,37]. In this direction, Köhler and co-workers prepared and comparatively studied the catalytic activity of Pd/Al_2_O_3_, Pd/TiO_2_, Pd/NaY, and Pd/CeO_2_ [38]. The catalysts were able to efficiently catalyse Suzuki cross-coupling reactions and the obtained TOFs for each catalyst were: Pd/Al_2_O_3_ = 9600 h^−1^, Pd/TiO_2_ = 9700 h^−1^, Pd/NaY = 4100, and Pd/CeO_2_ = 4100 h^−1^. The Pd/Al_2_O_3_ and Pd/TiO_2_ catalysts showed very similar and high catalytic activities, while the activities of Pd/NaY and Pd/CeO_2_ were much lower. The TOF values of Pd/NaY and Pd/CeO_2_ catalysts are almost half of those obtained with Pd/Al_2_O_3_ and Pd/TiO_2_. These results clearly suggest that the nature of the support has a great influence on the activity of the catalyst.

**Figure 2:**
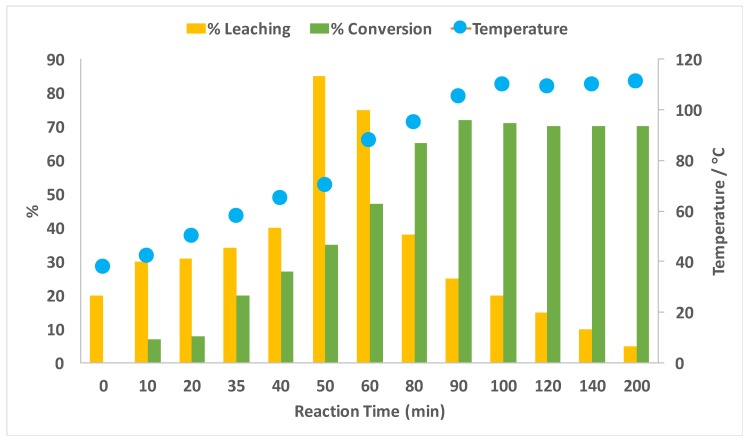
Time-dependent correlation of palladium leaching with the progress of the reaction [38] (Adapted with permission from John Wiley and Sons).

In addition, Köhler et al. stated that the Pd concentration in solution during the reaction correlated clearly with the progress of the reaction (Figure 2). Thus, this clearly suggests that the dissolved molecular palladium represents the “true” catalytically active species, and the prepared Pd/M_x_O_y_ solids are simply precursors to the “true catalyst”. Furthermore, they found that the dissolved “Pd is deposited back” onto the support at the end of the reaction. This phenomenon is commonly referred to as the palladium “release-capture or dissolution-redeposition” mechanism [39].

**Figure 3:**
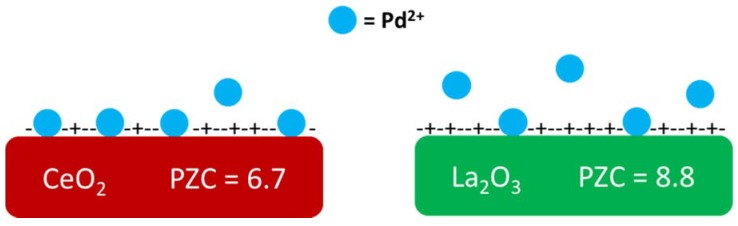
Representation of adhesion and release of Pd^2+^ from CeO_2_ and La_2_O_3_ surfaces under catalytic conditions (pH ~ 10.5) [42] (Adapted with permission from The Royal Society of Chemistry).

In 2013, Amoroso et al. [10] extended the study of Köhler and co-workers [38] to examine the catalytic activity of Pd/M_x_O_y_ type catalysts in more detail. More specifically, they wanted to investigate the influence of the “point of zero charge” on the catalytic activity of the Pd/M_x_O_y_ type catalysts. The point of zero charge (PZC) is commonly used in surface science to determine the ability of a given solid material to adsorb ions. This concept describes the condition when the electrical charge density on a surface of a solid is zero [40]. Generally, the PZC is represented as the pH value at which a solid submerged in an electrolyte exhibits zero net electrical charge on the surface [40,41]. A given metal oxide (M_x_O_y_) surface will have a “positive charge” at solution pH values less than the PZC value and thus be a surface on which anions may adsorb. On the other hand, the metal oxide surface will have a negative charge at solution pH values greater than the PZC value and thus be a surface on which cations may adsorb [40,41]. The later statement is more relevant to the current discussion since the pH of the reaction mixture in most cross-coupling reactions is around 10, thus it is above the PZC values of the investigated metal oxide supports (Figure 3). This allows one to investigate the degree of adhesion of palladium onto the support, which in turn speaks to the observed catalytic activity and the degree of palladium leaching.

Hence, Amoroso et al. [10] used the PZC values of each support to investigate the role it plays in the adhesion of the palladium nanoparticle onto the surface of the support (Figure 3). Thus, they prepared and investigated the catalytic activity of Pd/La_2_O_3_, Pd/Pr_6_O_11_, Pd/Gd_2_O_3_, Pd/Sm_2_O_3_, and Pd/CeO_2_ catalysts. The physicochemical properties of these materials are tabulated in Table 3. The study revealed that under basic reaction conditions (pH ~ 10.5), the surfaces of metal oxides with high PZC values are less negatively charged than the surfaces of metal oxides with low PZC values. Thus, the La_2_O_3_ support has the lowest density of negatively charged sites on the surface since it has the highest PZC value. Hence, the higher reactivity of Pd/La_2_O_3_ is correlated to a marked degree of Pd^2+^ leaching from the surface of La_2_O_3_, favoured by its less negatively charged surface. On the contrary, the lowest activity observed with Pd/CeO_2_ is correlated to the higher degree of negatively charged surface that helps in keeping Pd^2+^ ions anchored onto the support (Figure 3).

**Figure 4:**
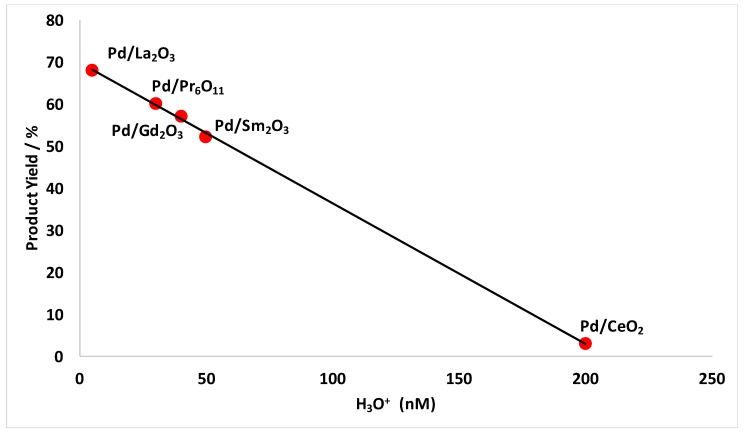
Cross-coupling reaction yield (after 2 min of reaction) vs. proton molar concentration (10^−PZC^) for Pd/M_x_O_y_ [10] (Adapted with permission from Springer Nature).

Figure 4 further shows that there is a linear correlation between the yield of the coupling product and the PZC ([H_3_O^+^] = 10^−PZC^) value of the metal oxide support. Thus, the observed reactivity trend over the M_x_O_y_ support is related to the tendency of each Pd/M_x_O_y_ system to deliver different amounts of palladium into the solution. In summary, the above discussion shows that there is a close relation between the “surface charge” (correlated to the PZC value) and the extent of palladium leaching at the pH of catalysis (determined by reaction conditions). Thus, the more positively charged is the surface (this is the case of Pd/La_2_O_3_), the more consistently Pd leaches, and therefore, a higher reaction rate is observed. In addition, a much slower reaction is observed when Pd/CeO_2_ is used as the catalyst, which shows the lowest PZC value within the series of Pd/M_x_O_y_. Consequently, prolonged recycling is possible in the case of Pd/CeO_2_ due to minimal palladium leaching. Thus, from the studied series of Pd/M_x_O_y_ type catalysts, Pd/CeO_2_ was chosen as superior precatalyst based on the following considerations: (i) it can be recycled several times without a significant decrease of activity (prolonged recyclability) and (ii) the organic product is sparsely contaminated since there is minimal leaching of palladium into the solution.

**Figure 5:**
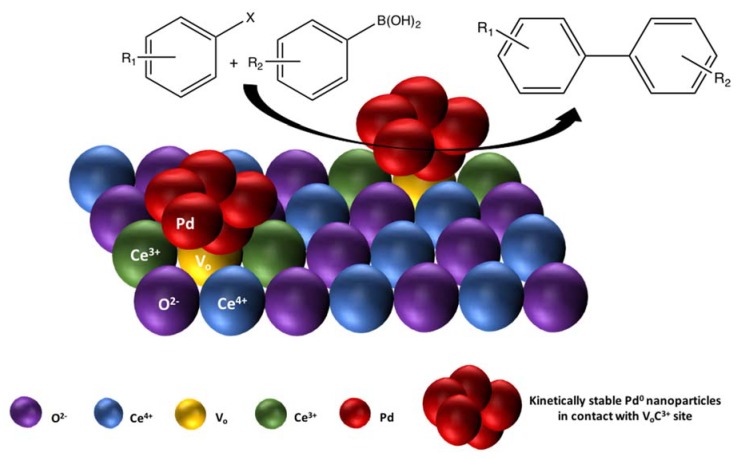
Plausible heterogeneous catalytic reaction mechanism suggested for Suzuki cross-coupling over the Pd/CeZrO_4-δ_ redox catalyst [52] (Adapted with permission from John Wiley and Sons).

Alternatively, the physicochemical properties of the support can be enhanced through substitution of a foreign metal ion in its lattice structure [43]. The substitution of foreign metal ions into the lattice of reducible oxides such as CeO_2_ and TiO_2_ to form ionic solid-solution oxides (Ce_1-x_M_x_O_2-δ_ or Ti_1-x_M_x_O_2-δ_) has given materials which show high activity for a variety of catalytic reactions, especially in gas-phase oxidation reactions [11,43,44,45,46,47,48]. However, such catalysts have rarely been applied to C–C cross-coupling reactions [49,50,51]. Recently, Burange and co-workers [44] reported that the ionic ceria-zirconia (CeZrO_4-δ_) solid-solution oxides exhibit high redox properties and thermal stability that make them better catalyst supports than the pure metal oxide counterparts [52]. Hence, CeZrO_4-δ_ solid-solution oxide was used to immobilise palladium nanoparticles and the resultant catalyst (Pd/CeZrO_4-δ_) was explored in Suzuki cross-coupling reactions (Figure 5). The mechanistic investigation proved that the redox couple (Ce^4+^/Ce^3+^) in the CeZrO_4-δ_ support enhances the catalytic activity through creation of oxygen vacancies. Furthermore, the support displayed strong metal-support (Pd-CeZrO_4-δ_) interactions and hence, “no leaching” was observed. Thus, it was concluded that the reactions were “truly” heterogeneous [52].

**Figure 6:**
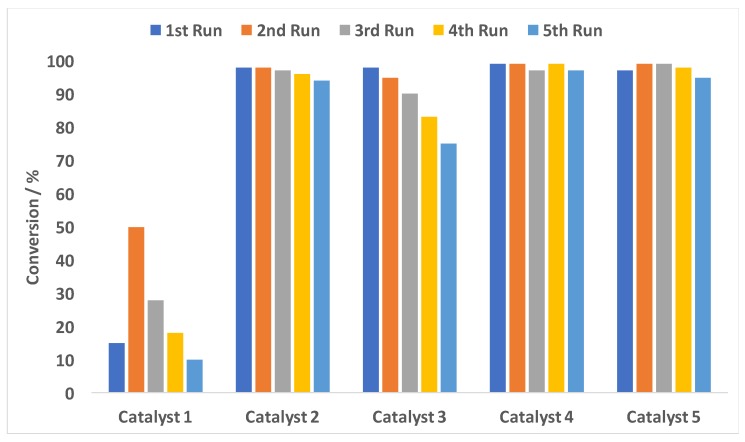
The Suzuki coupling of 4-bromotoluene with phenylboronic acid (after 30 min) using 0.5 mol % Pd. **Catalyst 1:** Ce_0.99_Pd_0.01_O_2-δ_, **Catalyst 2:** Ce_0.79_Sn_0.2_Pd_0.01_O_2-δ_, **Catalyst 3:** Ce_0.495_Sn_0.495_Pd_0.01_O_2-δ_, **Catalyst 4:** Ce_0.20_Sn_0.79_Pd_0.01_O_2-δ_, and **Catalyst 5:** Sn_0.99_Pd_0.01_O_2-δ_ [53] (Adapted with permission from Elsevier).

Lichtenegger and co-workers [53] extended the Burange et al. [52] study by directly incorporating palladium ions into the lattice of the support (ceria), instead of modifying the properties of the support with a foreign metal. In their study, they used CeO_2_ and SnO_2_ as supports and prepared a series of corresponding ionic solid-solution oxides: Ce_0.99_Pd_0.01_O_2-δ_, Ce_0.79_Sn_0.2_Pd_0.01_O_2-δ_, Ce_0.495_Sn_0.495_Pd_0.01_O_2-δ_, Ce_0.20_Sn_0.79_Pd_0.01_O_2-δ_, and Sn_0.99_Pd_0.01_O_2-δ_. These catalysts were thoroughly explored in Suzuki coupling reactions of phenylboric acid with various bromoarenes (Figure 6). The Ce_0.79_Sn_0.2_Pd_0.01_O_2-δ_, Ce_0.20_Sn_0.79_Pd_0.01_O_2-δ_, and Sn_0.99_Pd_0.01_O_2-δ_ catalysts showed extraordinarily high activities in Suzuki cross-coupling reactions, while the binary solid-solution oxide Ce_0.99_Pd_0.01_O_2-δ_ proved to be the least active catalyst. Thus, the Sn containing catalysts were shown to be more active; however, the results do not show a clear correlation between Sn loadings and the catalytic activity. Furthermore, complete conversion was achieved in five subsequent reactions for all catalysts, except when Ce_0.99_Pd_0.01_O_2-δ_ and Ce_0.495_Sn_0.495_Pd_0.01_O_2-δ_ were used.

**Figure 7:**
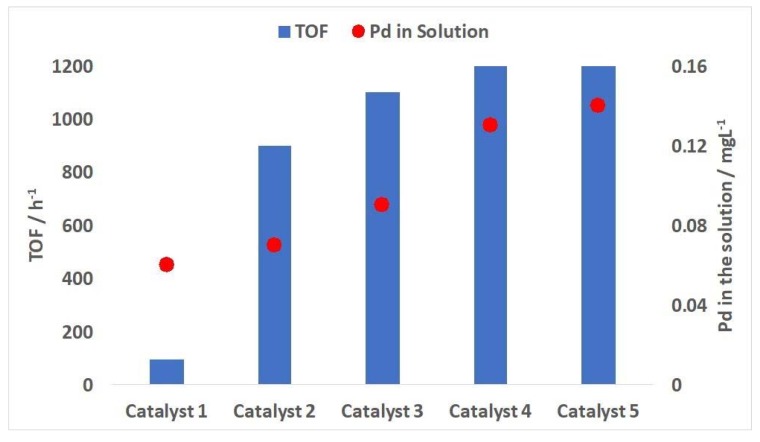
Turnover frequency (TOF) vs. Pd-concentration in the solution (Pd leached). **Catalyst 1:** Ce_0.99_Pd_0.01_O_2-δ_, **Catalyst 2:** Ce_0.79_Sn_0.2_Pd_0.01_O_2-δ_, **Catalyst 3:** Ce_0.495_Sn_0.495_Pd_0.01_O_2-δ_, **Catalyst 4:** Ce_0.20_Sn_0.79_Pd_0.01_O_2-δ_, and **Catalyst 5:** Sn_0.99_Pd_0.01_O_2-δ_ [53] (Adapted with permission from Elsevier).

Thorough catalyst leaching, recovery and recyclability studies were conducted and the results demonstrate a clear correlation between reactivity and amount of leached palladium (Figure 7). Hence, these findings also support the hypothesis that the coupling reaction is catalyzed by small amounts of leached palladium via a homogeneous reaction mechanism.

**Scheme 4:**
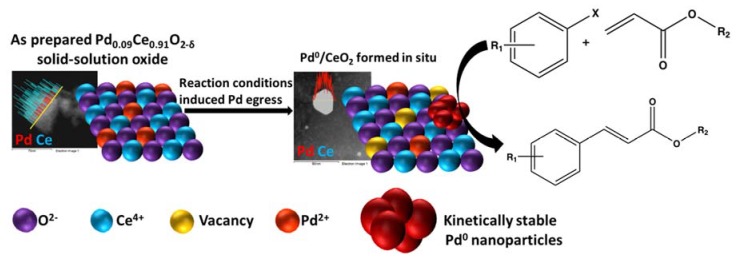
Proposed Heck coupling reaction mechanism catalysed by the Pd_0.09_Ce_0.91_O_2-δ_ precatalyst [49] (Adapted with permission from Elsevier).

To further explore this concept of “release-capture mechanism” by ionic solid-solution oxides catalysts, we have reported on the application of a highly Pd substituted ceria catalyst (Pd_0.09_Ce_0.91_O_2-δ_) in the Heck cross-coupling reaction [49]. Good to excellent yields of substituted olefins were obtained with this Pd_0.09_Ce_0.91_O_2-δ_ solid-solution catalyst. However, our findings also revealed that the catalysis occurs over the reduced two phase Pd^0^/CeO_2_ and not on the as prepared monophasic Pd_0.09_Ce_0.91_O_2-δ_ (Scheme 4). In addition, it was observed that the Pd^0^/CeO_2_ formed in situ can be easily recovered and reused for several times without any significant loss in efficiency and only a negligible amount of palladium was detected in the product solution (0.35 ppm). The low level of palladium concentration in the product solution further highlights the excellent ability of ceria to re-adsorb leached palladium species, as discussed earlier.

**Scheme 5:**
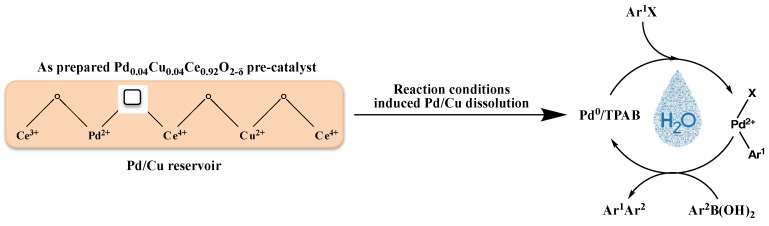
A proposed reaction mechanism for the Pd_0.04_Cu_0.04_Ce_0.92_O_2-δ_ (PdCuCeO)- tetrapropylammonium bromide (TPAB) catalysed quasi-heterogeneous SM (Suzuki-Miyaura) cross-coupling reactions [50].

We were also able to show that the reaction conditions greatly affect the catalysis. In this regard, we investigated the application of a Pd_0.04_Cu_0.04_Ce_0.92_O_2-δ_ (PdCuCeO) solid-solution oxide on Suzuki cross-coupling reactions (Scheme 5) [50]. The reactions were carried-out under milder and more environmentally friendly reaction conditions, using water as sole solvent and tetrapropylammonium bromide (TPAB) as a phase transfer catalyst. The catalytic results displayed good functional group tolerance and good to excellent yields of substituted biphenyls were obtained. However, catalyst leaching and recyclability studies revealed that PdCuCeO acts as a Pd reservoir and that the reactions were essentially quasi-heterogeneous, occurring over recoverable Pd/TPAB aggregates (generated in situ). The PdCuCeO solid-solution catalyst was recovered and recycled three times; however, its catalytic activity declined with each subsequent recycle.

Hence, it can be concluded that most of the reported ionic solid-solution oxides catalysts are simply precatalysts that act as a palladium reservoir for the active palladium species. Therefore, the observed high activity and recyclability of the catalysts could be attributed to a Pd release-capture mechanism, as discussed earlier.

## 5. Palladium Nanoparticle Immobilised on Magnetic Supports

**Figure 8:**
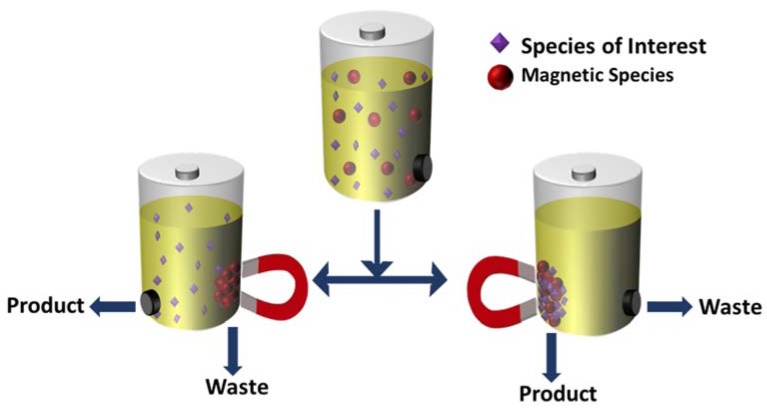
Examples of the use of magnetic separation through the application of an external magnetic field [63] (Adapted with permission from The Royal Society of Chemistry).

Recent studies show that magnetic nanoparticles are excellent supports for various catalysts [54,55,56,57,58,59,60,61]. Additionally, the magnetic properties can be exploited to improve catalyst separation from previous filtration and centrifugation methods. Supported palladium magnetic catalysts have the benefits of easy recovery from the reaction media by the application of an external magnetic field (Figure 8) [57]. Magnetic nanoparticles can be categorized into four main groups, namely; metals (Fe, Co, Ni), alloys (FePt, FePd), ferrites (CoFe_2_O_4_, CuFe_2_O_4_), and most notably metal oxides (FeO, Fe_2_O_3_, Fe_3_O_4_). Iron oxides are the most widely employed in literature because they have stronger magnetic properties and can be synthesized easily by co-precipitation methods [62].

A growing number of these magnetic supports have been applied in the development of magnetically recoverable palladium based heterogeneous catalysts for C–C cross-coupling reactions. Nasrollahzadeh and co-workers have explored the application of Pd/Fe_3_O_4_ magnetic nanoparticles as an efficient catalyst for C–C cross-coupling reactions [64]. The Pd/Fe_3_O_4_ magnetic catalyst was found to be stable during the Suzuki coupling reaction and good yields (83–95%) were obtained over a wide range of substrates. At the end of the Suzuki reaction the catalyst was recovered by the application of an external magnet, and successively reused five times without significant loss of activity.

**Figure 9:**
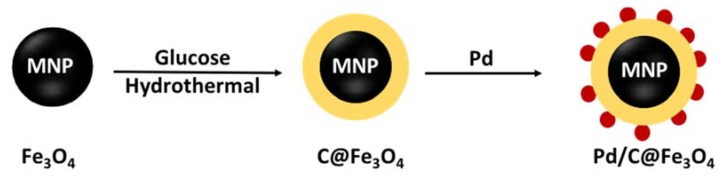
Schematic illustration of synthesis steps for C@Fe_3_O_4_ core-shell nano-spheres [66] (Adapted with permission from Elsevier).

**Figure 10:**
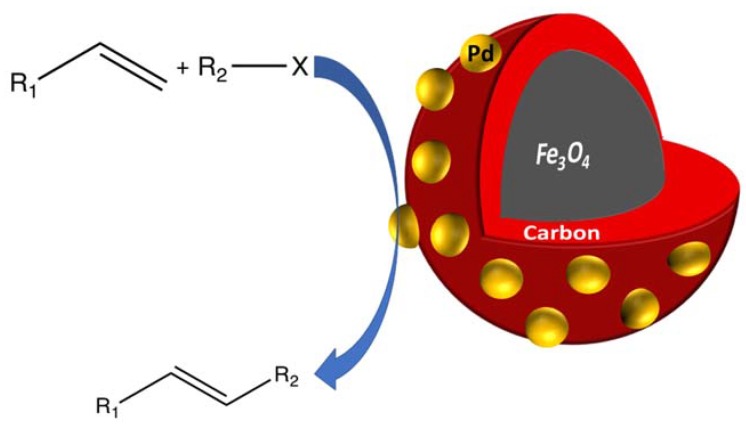
Graphical illustration of Pd/C@Fe_3_O_4_ catalysed cross-coupling reactions [66] (Adapted with permission from Elsevier).

Although bare or “naked” iron oxide nanoparticles have been shown to be efficient supports for cross-coupling reactions, the immobilization of palladium directly on the iron oxide occasionally suffers from aggregation or surface oxidation. The oxidation and agglomeration of magnetic nanoparticles results in the loss of their magnetism; thus, it is crucial to develop efficient strategies to strengthen their chemical stability [60]. In this regard, a wide range of stabilizing or coating materials, including organic stabilizers (polymers and surfactants) and inorganic stabilizers (silica and carbon materials), have been used to protect magnetic nanoparticles [60,65]. In this direction, Kumar et al. [66] developed efficient C@Fe_3_O_4_ magnetic core-shell nano-spheres by coating the Fe_3_O_4_ nanoparticle with a carbon shell to protect them from being corrupted or oxidized under cross-coupling reaction conditions. Palladium was then immobilised on the synthesized C@Fe_3_O_4_ magnetic core-shell to form a structurally stable Pd/C@Fe_3_O_4_ catalyst (Figure 9 and Figure 10). This catalyst was efficiently used as a heterogeneous catalyst for Heck cross-coupling reactions and good to excellent yields were obtained. The catalyst was easily separated from the reaction mixture through application of an external magnet and the catalyst was reused five times without any significant drop in activity. The authors claim that this catalytic system is “truly heterogeneous” even though some leaching was observed. They concluded that their catalytic system is advantageous because of its heterogeneity, high stability, gram scale applicability, magnetic separability, and consequent reusability [66].

**Figure 11:**
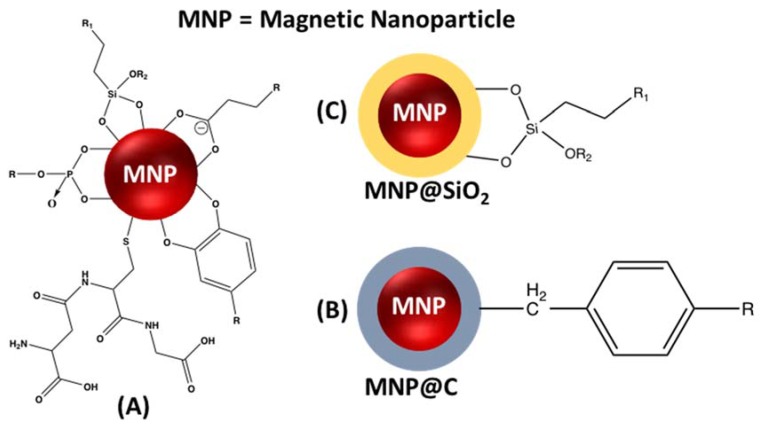
Different approaches to functionalize magnetic nanoparticles (MNPs): (**A**) iron oxide MNPs; (**B**) silica-coated MNPs; and (**C**) carbon-coated MNPs [63] (Adapted with permission from The Royal Society of Chemistry).

The coated magnetic nanoparticles can be further modified by functionalising their surface with various functional groups (Figure 11). The functionalization of the surface of magnetic nanomaterials is an important step in the design of supported catalysts, because it significantly improves the physical and chemical properties of the magnetic supports [63].

**Figure 12:**
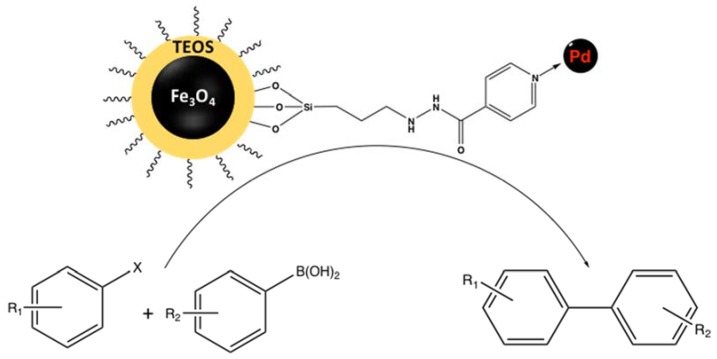
Fe_3_O_4_@SiO_2_/isoniazide/Pd mediated Suzuki cross-coupling reaction [67] (Adapted with permission from Elsevier).

Heidari and co-worker [67] used isoniazide to functionalize the surface of the Fe_3_O_4_@SiO_2_ magnetic nano-support (Figure 12). The isoniazide groups were used as linkers to immobilize palladium nanoparticles and prevent agglomeration on the surface of the Fe_3_O_4_@SiO_2_ magnetic nano-support [67]. The resulting material, Fe_3_O_4_@SiO_2_/isoniazide/Pd catalyst, was explored as a heterogeneous catalyst in Suzuki coupling reactions and good to excellent yields were obtained. The authors reported that the benefits of their heterogeneous catalyst system were its high efficiency and reuse was easily achieved through the use of a magnet. They were able to recover and recycle their catalyst six times without any noticeable loss in activity.

**Figure 13:**
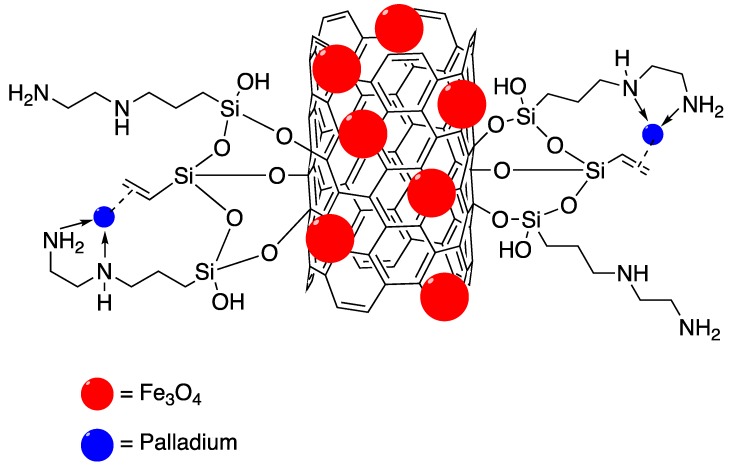
A graphical illustration of the CNT@Fe_3_O_4_@SiO_2_-Pd catalyst [12] (Adapted with permission from Springer Nature).

An interesting but slightly different approach was reported by Khalili and co-workers [12], who supported palladium nanoparticles on an amino-vinyl silica functionalized magnetic carbon nanotube (CNT). Their core–shell contains CNT@Fe_3_O_4_@SiO_2_-Pd in which the functionalized SiO_2_ helps with the stability of the Pd nanoparticles and is also responsible for the reduction of Pd(II) to Pd(0) without the need for adding external reducing agents (Figure 13). This catalyst was successfully applied to Heck and Suzuki cross-coupling reactions and high yields were obtained. In addition, the catalyst exhibited good recyclability, and was used for six consecutive recycles without a significant loss in catalytic activity.

**Scheme 6:**
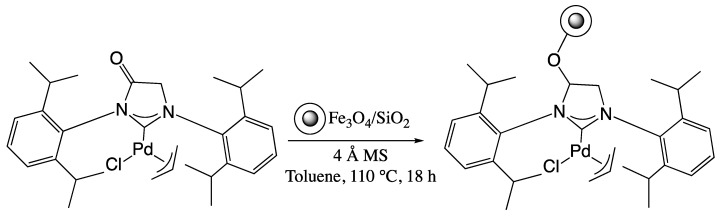
Immobilisation of the [*N*-heterocyclic carbene (NHC)Pd(allyl)Cl] complex on silica-coated magnetic nanoparticles [68] (Adapted with permission from Elsevier).

There are also numerous examples where organopalladium complexes, instead of palladium nanoparticles, have been immobilised on magnetic supports [68,69]. Recently, Collinson and co-workers [68] prepared a [(NHC)Pd(allyl)Cl] complex, bearing an *N*-heterocyclic carbene (NHC) and immobilised it on silica-coated magnetic nanoparticles (Scheme 6). Their strategy tries to combine the high activity of palladium–NHC catalysts with the facile separation and recyclability of a heterogeneous catalyst. The catalyst was then explored on Suzuki coupling reactions and good to excellent yields were obtained with a variety of substrates. However, the immobilised palladium complex could not be reused because the catalyst disassembled or decomposed under Suzuki coupling reaction conditions. The recycled catalyst only achieved 20% yield compared to the 90% yield that was obtained in its first use. The authors suspected that the base was responsible for the detachment of the magnetic silica linker.

**Figure 14:**
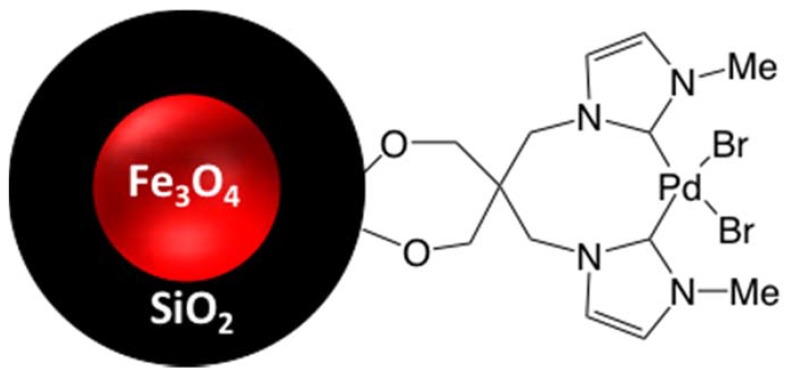
Bis(*N*-heterocyclic carbene) palladium complex supported on silica coated magnetic nanoparticles [69] (Adapted with permission from John Wiley and Sons).

Better results were obtained by Fareghi-Alamdari et al. [69] who supported bis(*N*-heterocyclic carbene) palladium complex on silica coated magnetic nanoparticles (Figure 14).

Their catalyst was efficiently used for Suzuki coupling reactions and at the end of the reaction, it was separated though application of an external magnet and recycled for six consecutive reactions. Hence, it not easy to identify the reaction parameters that caused the Collinson and co-workers [68] catalytic system to be unrecyclable because the reaction conditions are different (Table 4). However, the most obvious difference is the choice of the base; Collinson and co-workers used NaOH, which is very harsh, while Fareghi-Alamdari et al. used K_2_CO_3_. Hence, optimisation of reaction conditions is crucial in developing efficient and more robust catalytic systems.

In summary, the literature shows that magnetic nanoparticle-based catalytic systems have a huge potential in solving the recoverability and recyclability challenges [57].

## 6. Polymer Supported Palladium Nanoparticles

**Scheme 7:**
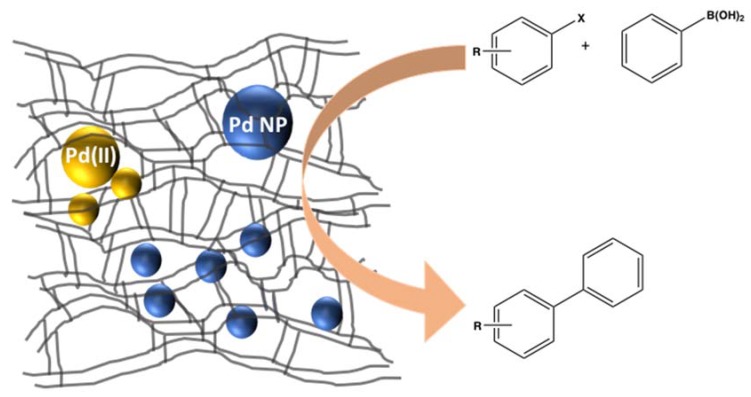
Catalysts for Suzuki cross-coupling based on functionalized hyper-crosslinked polystyrene [78] (Adapted with permission from American Chemical Society).

Literature reports have repeatedly shown that palladium nanoparticles can be efficiently supported on polymer frameworks [70,71,72,73,74,75,76,77]. Recently, Nemygina and co-workers immobilised various palladium precursors (PdCl_2_, PdCl_2_(CH_3_CN)_2_, and PdCl_2_(PhCN)_2_) on amino-functionalized hyper-crosslinked polystyrene [78]. They then evaluated the influence of palladium oxidation state (Pd(II) or Pd(0)) on the rate of Suzuki cross-coupling of 4-bromoanisole and phenylboronic acid. Their results revealed that the catalyst impregnated with a PdCl_2_(CH_3_CN)_2_ precursor resulted in better catalytic activity (Scheme 7). The PdCl_2_(CH_3_CN)_2_ precursor was believed to be more active because it experienced less hydrolysis and precipitation in comparison with PdCl_2_ and PdCl_2_(PhCN)_2_ under Suzuki coupling reaction conditions. Lastly, they observed that unreduced, Pd(II) containing pre-catalyst were more active [78].

**Scheme 8:**
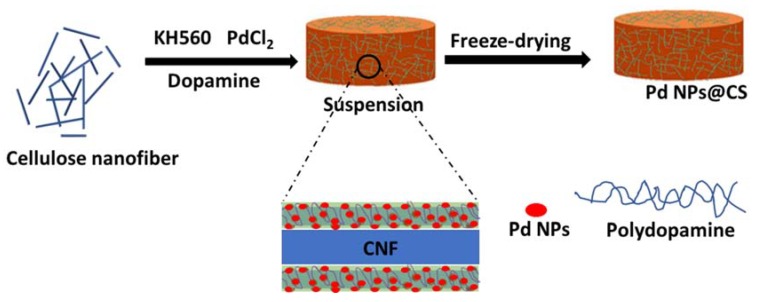
Schematic of the stepwise formation of cellulose sponge supported palladium nanoparticles [79] (Adapted with permission from American Chemical Society).

Another interesting strategy for immobilising palladium on a polymer was reported by Li and co-workers [79], who synthesised a robust and flexible cellulose sponge using a dual-cross-linking cellulose nanofiber (CNF) with γ-glycidoxypropyltrimethoxysilane (GPTMS) and polydopamine (PDA) (Scheme 8). The characterisation results revealed that the palladium nanoparticles were homogeneously dispersed on the surface of the cellulose nanofiber with a narrow size distribution. The catalyst was successfully applied to heterogeneous Suzuki and Heck cross-coupling reactions. Leaching of palladium was negligible and the catalysts could be conveniently separated from the products and reused for six times without loss of activity [79].

## 7. Palladium Nanoparticles Immobilized on Hybrid Inorganic-Organic Material

**Figure 15:**
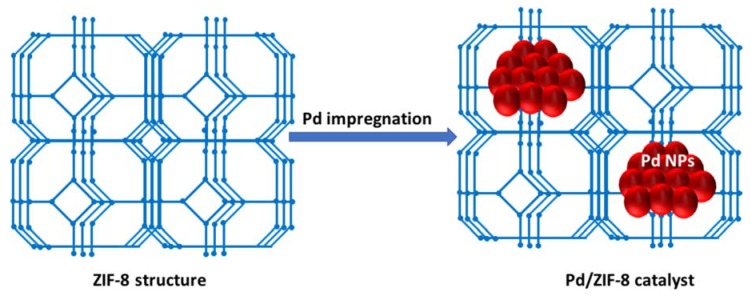
A graphical illustration of the Pd/zeolitic imidazolate (ZIF-8 catalyst) [88] (Adapted with permission from John Wiley and Sons).

The hybrid inorganic–organic materials are crystalline systems in which both inorganic and organic structural elements co-exist within a single phase [80]. These hybrid inorganic–organic materials are gaining popularity in cross-coupling reactions due to their tunable pore size, high surface areas, high crystallinity, and structural diversity [81,82,83,84,85,86,87]. Most hybrid inorganic–organic materials are synthesised through functionalization of the support surface with appropriate functional groups. This allows for a better control of the dimensions, dispersion and stability of the palladium nanoparticles. Metal-organic frameworks (MOFs) are one of the most popular classes of hybrid inorganic–organic materials and they are frequently explored as suitable palladium supports in heterogeneous cross-coupling reactions. For example, Shaikh and co-workers [88] used a zeolitic imidazolate framework (ZIF) to develop robust ZIF-supported palladium nanoparticles as a heterogeneous catalyst for Heck cross-coupling reactions (Figure 15). The Pd/ZIF catalyst was found to be highly efficient and could be re-used up to four times without any loss in catalytic efficiency [88].

Another interesting example was reported by Kozell et al. [89] who immobilised palladium nanoparticles on zirconium phosphate glycine diphosphonate nanosheets (ZPGly). The immobilization of palladium nanoparticles on ZPGly nanosheets provided a stable catalyst with high palladium content (up to 22 wt. %). This Pd@ZPGly-15 catalyst proved to be highly efficient in catalysing Suzuki and Heck cross-couplings reactions. Surprisingly, the authors reported that their catalytic system operates as an efficient “heterogeneous” system, even though 3–5 ppm of palladium leached (Table 5). In addition, a “release and catch” mechanism was proposed in which soluble active palladium species are released from the solid Pd@ZPGly-15 catalyst during the oxidative addition of the halide and then re-deposited on the ZPGly support as a consequence of the reductive elimination step at the end of the reaction [89].

**Figure 16:**
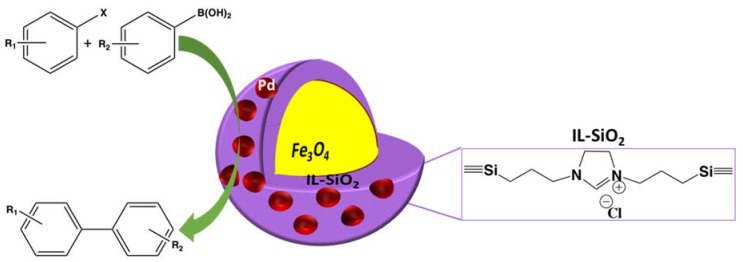
Suzuki cross-coupling reactions catalysed by the Pd/MNP@IL-SiO_2 _(palladium nanoparticles on the organic-silica nanoparticles) catalyst [90] (Adapted with permission from American Chemical Society).

A magnetically separable version of a hybrid organic-inorganic support was recently reported by Omar and co-workers [90]. They supported palladium on magnetically separable organic-silica hybrid nanoparticles that were functionalized with ionic liquid groups (Figure 16). The presence of ionic liquid groups within the framework of the hybrid nanoparticles enhanced the stability of the palladium nanoparticles on the organic-silica nanoparticles. The resultant Pd/MNP@IL-SiO_2_ catalyst was efficiently applied in Heck and Suzuki cross-coupling reactions and it demonstrated high catalytic activity (Figure 16). The Pd/MNP@IL-SiO_2_ catalyst was also easily separated from the reaction mixture by application of an external magnetic field and the catalyst could be recycled over five times without a significant loss in its catalytic activity. In conclusion, the authors envisaged that this and similar catalytic systems could pave a way for the desired bridging of homogeneous and heterogeneous catalysis.

**Scheme 9:**
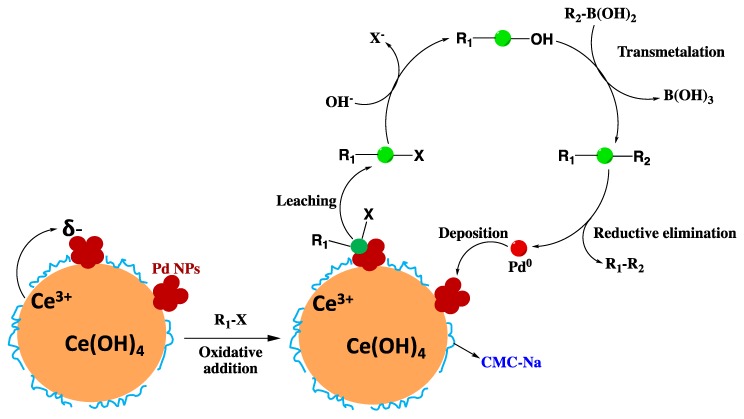
Illustrative diagram of the conceptual mechanism of the Suzuki reaction catalyzed by Pd/CMC@Ce(OH)_4_ [91] (Adapted with permission from Elsevier).

Another special type of hybrid organic-inorganic support was reported by Lin et al. who prepared a retrievable Pd/CMC@Ce(OH)_4_ catalyst for Suzuki coupling-reactions [91]. In this system, Ce(OH)_4_ was coated with carboxymethylcellulose (CMC) to form a hybrid organic-inorganic CMC@Ce(OH)_4_ support (Scheme 9). Carboxymethylcellulose has a large number of inherent carboxylate (–COO−) and hydroxyl (–OH) groups on its molecular chain. Thus, CMC is an excellent cation exchange agent, which makes it an excellent support for stabilization of palladium nanoparticles. While ceria has a unique ability of forming oxygen vacancies, that reduce Ce^4+^ to Ce^3+^ and create an excess of negative charge on its surface. The excess negative charge facilitates the coordination of palladium nanoparticles to the surface, as discussed earlier. These interactions have been shown to be crucial in preventing leaching and agglomeration of catalytic palladium nanoparticles. Scheme 9 shows an illustrative diagram of the conceptual mechanism of the Suzuki reaction catalyzed by Pd(0)–CMC@Ce(OH)_4_. The Ce^3+^ on the surface of Ce(OH)_4_ enables the electronic charge transfer that produces a highly negatively charged palladium center. This then makes palladium a more electron rich site for facile oxidative addition of aryl halides, and thus allows catalysis to commence.

The Pd/CMC@Ce(OH)_4_ catalyst was applied to the Suzuki cross-coupling reaction and good to excellent yields were obtained. It is believed that the activity of the catalyst was enhanced synergistically by the unique redox properties of Ce(OH)_4_ (Ce^3+^/Ce^4+^) and the coordination with the functional (carboxyl and hydroxyl) groups of the hybrid CMC@Ce(OH)_4_ support. Moreover, the catalyst could be easily separated by simple filtration and reused at least for five times without losing its activity. However, it was observed that Pd(0) species leached from the hybrid support during the reaction and were re–deposited onto the support at the end of the reaction. Therefore, the reaction mechanism is homogeneous in nature and the leached palladium species are the “true active species”.

## 8. Conclusions

In conclusion, the literature survey shows that remarkable progress has been made regarding the activity and recyclability of heterogeneous palladium based catalytic systems for C–C cross coupling reactions. However, thorough leaching and recyclability tests are still needed for most reported heterogeneous catalytic systems to unequivocally confirm that the palladium supported catalysts are responsible for the observed catalytic activity and not the leached material. Hence, at the moment it is hard to say what the nature of the true catalyst is, due to the dynamic nature of the reported catalytic systems for cross-coupling reactions.

## Figures and Tables

**Table 1:** Maximum acceptable concentration limits for the residues of metal catalysts that can be present in pharmaceutical products [4].

Metal	Concentration (ppm)	
	Oral	Parenteral
Pd, Pt, Ir, Rh, Ru, Os	5	0.5
Mo, V, Ni, Cr	10	1.0
Cu, Mn	15	1.5
Zn, Fe	20	2.0

**Table 2:** Performance of PdO supported carbon materials in the Heck coupling reaction [32] (Adapted with permission from John Wiley and Sons).

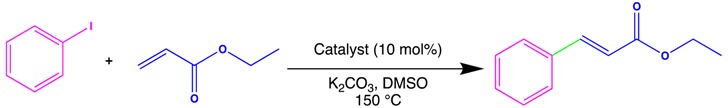			
Catalyst ^a^	Reaction Time (min)	Yield (%) ^b^	TOF (h^−1^)
PdO/SWCNT	20	72	939
PdO/MWCNT	15	63	1080
PdO/CNF	40	38	274
PdO/GO	5	80	4144
PdO/RGO	90	68	194

^a^ Reaction Conditions: Iodobenzene (0.25 mmol), ethyl acrylate (0.5 mmol), PdO/support (10 mol %), K_2_CO_3_ (0.5 mmol), DMSO (dimethylsuloxide) (1 mL) at 150 °C. ^b^ GC yields.

**Table 3:** The physicochemical properties of the synthesised Pd/M_x_O_y_ based catalysts and their catalytic activity results [10] (Adapted with permission from Springer Nature).

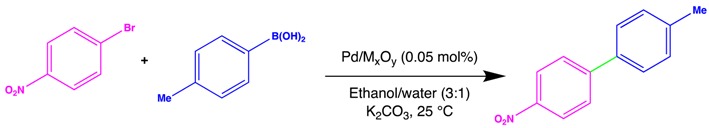						
Compound (Pd/M_x_O_y_) ^a^	Pd Loading (wt %)	Surface Area (m^2^/g)	Pore Volume (cm^3^/g)	PZC (pH)	Time (min)	TOF (h^−1^)
Pd/La_2_O_3_	1.76	16.6	0.24	8.8	13	9130
Pd/CeO_2_	1.93	31.5	0.18	6.7	260	450
Pd/Pr_6_O_11_	1.93	22.4	0.36	7.8	17	6980
Pd/Sm_2_O_3_	1.84	11.9	0.21	7.4	21	5650
Pd/Gd_2_O_3_	1.99	10.2	0.09	7.5	20	5940

^a^ Reaction conditions: 1-bromo-4-nitrobenzene (0.5 mmol), 4-methylphenylboronic acid (0.6 mmol), K_2_CO_3_ (0.6 mmol), catalyst, 1.5 mL ethanol, 0.5 mL water, temperature = 25 °C.

**Table 4:** The reaction conditions for Suzuki cross-coupling reactions catalysed by supported palladium complexes in references [68,69].

	Reaction Conditions		
	Solvent	Base	Temperature (°C)
Collinson et al. [68] ^a^	Isopropanol	NaOH	60
Fareghi-Alamdari et al. [69] ^b^	DMF/H_2_O	K_2_CO_3_	80

^a^ Reaction conditions: Bromotoluene (1.0 mmol), phenylboronic acid (1.05 mmol), NaOH (1.1 mmol), catalyst (1 mol % Pd), isopropanol (1 mL). ^b^ Reaction conditions: Bromotoluene (1.0 mmol), phenylboronic acid (1.2 mmol), K_2_CO_3_ (1.5 mmol), catalyst (0.12 mol % Pd), DMF/H_2_O (2:1, 3 mL).

**Table 5:** Suzuki cross-coupling reaction between 4-bromobenzaldehyde and phenylboric acid catalysed by Pd@ZPGly-15 (palladium nanoparticles on zirconium phosphate glycine diphosphonate nanosheets) catalyst [89].

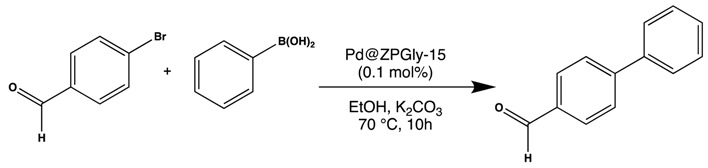		
Entry ^a^	Yield (%) ^b^	Pd Leaching (ppm)
Run 1	98	5
Run 2	98	3
Run 3	98	3

^a^ Reaction conditions: 4-Bromobenzaldehyde (1.0 mmol), phenylboronic acid (1.2 mmol), K_2_CO_3_ (1.1 mmol), catalyst (0.1 mol % Pd), ethanol (2.4 mL). ^b^ GC (gas chromatography) yields.
